# APDCA: An accurate and effective method for predicting associations between RBPs and AS-events during epithelial-mesenchymal transition

**DOI:** 10.1371/journal.pcbi.1013665

**Published:** 2025-11-06

**Authors:** Yangsong He, Zheng-Jian Bai, Wai-Ki Ching, Quan Zou, Yushan Qiu

**Affiliations:** 1 School of Mathematical Sciences, Shenzhen University, Shenzhen, People’s Republic of China; 2 School of Mathematical Sciences, Xiamen University, Fujian, People’s Republic of China; 3 Department of Mathematics, The University of Hong Kong, Hong Kong, People’s Republic of China; 4 Institute of Fundamental and Frontier Sciences, University of Electronic Science and Technology of China, Chengdu, People’s Republic of China; University of Notre Dame, UNITED STATES OF AMERICA

## Abstract

**Motivation:** Epithelial-mesenchymal transition (EMT) plays a key role in cancer metastasis by promoting changes in adhesion and motility. RNA-binding proteins (RBPs) regulate alternative splicing (AS) during EMT, enabling a single gene to produce multiple protein isoforms that affect tumor progression. Disruption of RBP-AS interactions may disrupt the progress of diseases like cancer. Despite the importance of RBP-AS relationships in EMT, few computational methods predict these associations. Existing models struggle in sparse settings with limited known associations. To improve performance, we incorporate both sparsity constraints and heterogeneous biological data to infer RBP–AS associations.

**Result:** We propose a new method based on Accelerated Proximal DC Algorithm (APDCA) for predicting RBP–AS associations. In particular, APDCA combines sparse low-rank matrix factorization with a Difference-of-Convex (DC) optimization framework and uses extrapolation to improve convergence. A key feature of APDCA is the use of a sparsity constraint, which filters out noise and highlights key associations. In addition, integrating multiple related data sources with direct or indirect relationships can help in reaching a more comprehensive view of RBPs and AS events and to reduce the impact of false positives associated with individual data sources. we prove that our proposed algorithm is convergent under some conditions and the experimental results have illustrated that APDCA outperforms six baseline methods in both AUC and AUPR. A case study on the RBP QKI shows that the top predictions are verified by the OncoSplicing database. Thus, APDCA provides a fast, interpretable, and scalable tool for discovering post-transcriptional regulatory interactions.

## Author summary

Epithelial–mesenchymal transition (EMT) is a fundamental biological process closely linked to cancer metastasis, where RNA-binding proteins (RBPs) play a critical role by regulating alternative splicing (AS) events. Accurately identifying RBP–AS associations during EMT is essential for understanding post-transcriptional regulation, yet existing computational approaches often struggle with the sparsity of known interactions and the heterogeneity of biological data. To address these challenges, we propose APDCA, an Accelerated Proximal Difference-of-Convex Algorithm, which integrates heterogeneous biological data and formulates the prediction task as a difference-of-convex optimisation problem. APDCA introduces a proximal optimisation scheme enhanced by Nesterov extrapolation, enabling both noise suppression and efficient convergence. The algorithm jointly enforces low-rank structure to capture shared regulatory patterns and sparsity constraints to filter out spurious associations. Moreover, it incorporates adaptive weight updating and error controlled regularisation, eliminating the need for pre-constructed similarity graphs or manual parameter tuning. This unified framework enables APDCA to learn biologically meaningful and interpretable associations with high computational efficiency. Through extensive experiments, APDCA demonstrates superior performance over six existing methods and rapidly identifies high-confidence RBP–AS pairs for downstream biological validation.

## 1 Introduction

Alternative splicing (AS) is a crucial post-transcriptional regulatory mechanism in eukaryotes that allows a single gene to produce multiple transcript isoforms by variably splicing precursor mRNA [1]. This process greatly enhances the diversity of the proteome. RNA-binding proteins (RBPs) play a central role in regulating AS by recognizing specific sequence motifs and influencing splice site selection, exon inclusion or skipping, and transcript stability [2,3].

AS has been extensively studied in various cellular contexts. However, its regulation during epithelial–mesenchymal transition (EMT) remains underexplored [4]. EMT is a process in which epithelial cells lose their adhesion properties and acquire migratory traits typical of mesenchymal cells [5]. This transition is important for cancer metastasis, and disruption of AS during EMT has a significant impact on tumor progression. RBPs are central to the regulation of AS during EMT, making their interaction with AS events crucial to understanding tumor progression [6]. Thus, understanding the relationship between RBPs and AS events during EMT is essential to uncovering new cancer therapeutic targets.

Studies have shown that disruption of RBP–AS event interactions is closely associated with various diseases, including cancers, neurodegenerative disorders, and immune dysfunction [7,8]. For example, the RNA-binding protein QKI modulates the alternative splicing of the NUMB gene in lung cancer by binding to specific RNA elements in its pre-mRNA. This regulation suppresses tumor cell proliferation and inhibits the activation of the Notch signaling pathway, highlighting a critical role of QKI-mediated splicing control in tumor suppression [9]. Similarly, PTBP1 regulates splicing isoforms of the PKM gene, thereby impacting the glycolytic pathway and playing a key role in multiple types of tumors [10]. These examples underscore the functional diversity and specificity of RBPs in AS regulation. Therefore, systematically identifying and predicting regulatory relationships between RBPs and AS events is critical for elucidating transcriptomic regulatory mechanisms and discovering novel biomarkers or therapeutic targets.

Although recent years have seen growing attention to the regulatory relationships between alternative splicing (AS) events and RNA-binding proteins (RBPs), computational methods specifically designed to predict such associations remain limited [11]. In contrast, numerous models have been widely developed to predict potential associations between lncRNAs and diseases [12–15]. These methods are generally based on universal molecular network modeling strategies with strong generalizability, making them suitable for exploring associations among other biological entities. Current mainstream association prediction algorithms can be broadly divided into four categories. The first category includes machine learning–based methods. Zeng et al. proposed SDLDA, which constructs feature vectors based on similarities between biological entities and applies a support vector machine (SVM) classifier to determine potential associations [16]. Lan et al. introduced LDAP, which employs label-guided linear discriminant analysis (LDA) to project features into a discriminative subspace, improving the separability between associated and non-associated pairs [17]. The second category consists of graph-based methods. Wu et al. developed GAERF, which constructs a heterogeneous graph and uses a graph autoencoder to learn node embeddings, followed by a random forest classifier to predict associations [18]. The third category comprises matrix factorization methods [19]. Fu et al. proposed MLFDA, which performs multi-view low-rank matrix factorization to capture latent features from multiple association matrices and integrates structural information from various perspectives to enhance expressiveness [20]. Lu et al. designed SIMCLDA, which incorporates similarity-based regularization into non-negative matrix factorization, encouraging similar entities to share latent features and thereby improving robustness and generalization, especially under sparse data conditions [21]. The fourth category includes network propagation methods. Gu et al. introduced GrwLDA, which applies a random walk with restart strategy to simulate information diffusion across a heterogeneous network and infer potential associations [22].

Existing computational models for molecular association prediction still have limitations when applied to the identification of regulatory relationships between RNA-binding proteins (RBPs) and alternative splicing (AS) events during EMT. Many machine learning methods rely on predefined similarity features and lack the ability to integrate heterogeneous biological data. Graph-based and network-based models often depend on known molecular interaction networks, which may not capture hidden or novel associations. Matrix factorization techniques have been widely used due to their effectiveness in latent structure discovery, but most are limited to modeling single-type associations and fail to leverage the complementary information from diverse biological entities. Furthermore, existing methods rarely incorporate sparsity constraints, which are important for improving model interpretability, reducing noise, and enhancing generalization to unseen data. Biologically, only a small subset of RBPs tends to interact with any given AS event. So enforcing sparsity better reflects this selective regulatory landscape.

To address these challenges, we propose APDCA (Accelerated Proximal DC Algorithm), a novel matrix factorization framework specifically designed for predicting RBP–AS event associations during the EMT process. Our model integrates multiple types of biological entities to form a heterogeneous molecular network, enabling a more comprehensive representation of the regulatory landscape. To highlight important associations and suppress irrelevant ones, we introduce a sparsity constraint on the reconstructed matrix. This results in a non-convex optimization problem, which we reformulate using Difference-of-Convex (DC) programming to ensure solvability [23]. In addition, APDCA leverages a proximal optimization framework to handle the sparsity-induced non-smoothness, which improves stability and ensures convergence in complex, high-dimensional spaces. Proximal methods are well suited for non-differentiable problems and enable efficient iterative updates by incorporating regularization through proximity operators [24]. To further accelerate convergence, we adopt a Nesterov extrapolation technique during the iterative optimization process [25]. These innovations collectively allow APDCA to achieve accurate, interpretable, and scalable predictions across complex biological systems.

To evaluate the model’s performance, we conducted experiments using 5-fold cross-validation on the constructed dataset. The results show that the proposed APDCA model consistently outperforms six representative baseline methods, achieving the highest scores in both AUC (Area Under the ROC Curve) and AUPR (Area Under the Precision-Recall Curve). To further assess the model’s applicability and biological interpretability, we performed a case study based on the OncoSplicing database [26]. Focusing on specific RNA-binding proteins such as QKI, we explored the top-ranked predictions in detail, demonstrating the model’s potential to uncover meaningful regulatory associations between RBPs and AS events.

## 2 Materials and methods

### 2.1 Problem formulation

Throughout this paper, we use the following notations. Let ${\mathbb{R}}^{m\times n}$ be the set of all m×n real matrices, and ${\mathbb{R}}^{n}={\mathbb{R}}^{n\times 1}$. Let ${\mathbb{R}}^{n}$ be equipped with the Euclidean inner product ⟨·,·⟩ and its induced norm ‖·‖. The superscripts “$\cdot^{-1}$”, “$\cdot^{†}$”, and “$\cdot^{\text{T}}$” stand for the inverse, the Moore-Penrose pseudoinverse, and the transpose of a matrix, respectively. Denote by $\|\;\cdot \;{\|}_{F}$ and $\|\;\cdot \;{\|}_{0}$ the Frobenius matrix norm and the $\ell_{0}$ norm (i.e., the number of non-zero entries of a vector or matrix). Let tr(·) and vec(·) be the trace of a square matrix and a column vector from a matrix by stacking its column vectors below one another, respectively. Let e be a column vector with all elements equal to one. Finally, A≥O or a≥0 means a matrix or a vector in which all the elements are equal to or greater than zero and a≤e means that e−a≥0.

The problem can be described as follows: suppose there are *m* types of molecules, either directly or indirectly related to RBPs or AS-events, and a collection of relational data sources *R*. Each data source *R*_*ij*_ represents the interaction between objects of the *i*-th and *j*-th types, where i,j∈{1,2,…,m}. Specifically, $R_{ij}\in {\mathbb{R}}^{n_{i}\times n_{j}}$ stores the inter-relation between *n*_*i*_ objects of the *i*-th type and *n*_*j*_ objects of the *j*-th type. Note that *R*_*ij*_ can be asymmetric. Additionally, the intra-relation of objects within the *i*-th type is captured by a constraint matrix $\Theta_{i}\in {\mathbb{R}}^{n_{i}\times n_{i}}$. Matrix factorization-based data fusion aims to decompose the data matrices $R\in {\mathbb{R}}^{\left(\sum_{i=1}^{m}n_{i})\times (\sum_{i=1}^{m}n_{i}\right)}$ or its sub-matrices, while being constrained by the corresponding sub-matrices of $\Theta \in {\mathbb{R}}^{\left(\sum_{i=1}^{m}n_{i})\times (\sum_{i=1}^{m}n_{i}\right)}$. The decomposition yields low-rank matrices that explore latent relationships both within the same type of objects and across different types. These low-rank matrices are subsequently used to reconstruct the target association matrix, *R*_*ij*_, for predicting new associations between objects of the *i*-th type (RBPs) and objects of the *j*-th type (AS-events).

### 2.2 Objective function and constraints of APDCA

Biological interactions among different types of entities often exhibit strong sparsity, since many potential interactions never manifest in practice while only a few are functionally relevant. By preserving only the top *k* largest elements in *G*_*i*_, the model automatically filters out negligible interaction strengths, thus focusing on the most meaningful signals. This element-level sparsity design not only reduces noise and spurious connections but also helps prevent overfitting, ultimately improving both the interpretability and robustness of the final predictions.

Hence, we consider the following optimization problem:

$$\left\{ \begin{array}{cc} \min_{\begin{array}{c} S_{ij}\in {\mathbb{R}}^{k_{i}\times k_{j}},1\leq i,j\leq m\\ G_{i}\in {\mathbb{R}}^{n_{i}\times k_{i}},1\leq i\leq m \end{array}} & F(S,G):=\sum_{i,j}\omega_{ij}\|R_{ij}-G_{i}S_{ij}G_{j}^{\text{T}}{\|}_{F}^{2}\\ & \;\;+\lambda \sum_{r=1}^{\max_{i}r_{i}}\sum_{i=1}^{m}\text{tr}(G_{i}^{\text{T}}\Theta_{i}G_{i})\\ \text{subject to (s.t.)} & G_{i}\geq O,\|G_{i}{\|}_{0}\leq k,i=1,\ldots ,m, \end{array}\right.$$ (1)

where $\{\omega_{ij}>\;0{\}}_{i,j=1}^{m}$ are the prescribed weight parameters, and λ>0 is a regularized parameter.

$G_{i}\in {\mathbb{R}}^{n_{i}\times k_{i}}$ denotes the low-rank representation matrix for objects of the *i*-th type, and $S_{ij}\in {\mathbb{R}}^{k_{i}\times k_{j}}$ encodes the latent interactions between the *i*-th and *j*-th types. Multiplying *G*_*i*_, *S*_*ij*_, and $G_{j}^{\text{T}}$ reconstructs the observed matrix *R*_*ij*_ in a compact, low-dimensional form.

For any $x=(x_{i})\in {\mathbb{R}}^{n}$ and 1≤k≤n, let ${\left|\;|\;|x|\;|\;\right|}_{k}:=\sum_{t=1}^{k}|x_{\pi (t)}|$ be the largest-*k* norm of x, where *π* is a permutation of [n]:={1,2,…,n} such that $\left|x_{\pi (1)}|\geq |x_{\pi (2)}|\geq \ldots \geq |x_{\pi (n)}\right|$. Then we have


$$\|x{\|}_{0}\leq k\;\text{if and only if}\;{\left|\;|\;|x|\;|\;\right|}_{n}-{\left|\;|\;|x|\;|\;\right|}_{k}=0.$$

In particular, when x≥0,


$$\|x{\|}_{0}\leq k\;\text{if and only if}\;e^{T}x-s_{\left(k\right)}(x)=0,$$

where $s_{\left(k\right)}(x)=\sum_{t=1}^{k}x_{\pi (t)}$ be the sum of the *k* largest entries of x, where *π* is a permutation of [*n*] such that $x_{\pi (1)}\geq x_{\pi (2)}\geq \ldots \geq x_{\pi (n)}$. We note that the subdifferential of ${\left|\;|\;|\cdot |\;|\;\right|}_{k}$ at a point $x\in {\mathbb{R}}^{n}$ is given by [27,28]


$$\partial {\left|\;|\;|x|\;|\;\right|}_{k}=\arg\max_{z\in {\mathbb{R}}^{n}}\{\langle x,z\rangle \;|\;\sum_{t=1}^{n}|z_{i}|=k,-1\leq z_{i}\leq 1,i=1,\ldots ,n\}$$

and the subdifferential of $s_{\left(k\right)}(\cdot )$ at a point $x\in {\mathbb{R}}^{n}$ is given by [29]


$$\partial s_{\left(k\right)}(x)=\arg\max_{z\in {\mathbb{R}}^{n}}\{\langle x,z\rangle |\sum_{t=1}^{n}z_{i}=k,0\leq z_{i}\leq 1,i=1,\ldots ,n\}.$$

As noted in [29], for any $x=(x_{i})\in {\mathbb{R}}^{n}$ and 1≤k≤n, we can compute a subgradient $z\in \partial s_{\left(k\right)}(x)$ as follows: (i) Sort the entries of x in decreasing order, i.e., $x_{\pi (1)}\geq x_{\pi (2)}\geq \ldots \geq x_{\pi (n)}$; (ii) Set $z_{\pi (i)}=1$ for i=1,…,k and $z_{\pi (i)}=0$ for i=k+1,…,n. Analogiously, we can compute a subgradient $z\in \partial {\left|\;|\;|x|\;|\;\right|}_{k}$ as follows: (i) Sort the entries of **x** such that $\left|x_{\pi (1)}|\geq |x_{\pi (2)}|\geq \ldots \geq |x_{\pi (n)}\right|$; (ii) Assign $\text{sign}(x_{\pi (i)})$ to $z_{\pi (i)}$ for i=1,…,k and 0 to $z_{\pi (i)}$ for i=k+1,…,n.

From the above analysis, we see that problem Eq (1) is equivalent to the following DC-constrained problem:

$$\left\{ \begin{array}{cc} \min_{\begin{array}{c} S_{ij}\in {\mathbb{R}}^{k_{i}\times k_{j}},1\leq i,j\leq m\\ G_{i}\in {\mathbb{R}}^{n_{i}\times k_{i}},1\leq i\leq m \end{array}} & F(S,G):=\sum_{i,j}\omega_{ij}\|R_{ij}-G_{i}S_{ij}G_{j}^{\text{T}}{\|}_{F}^{2}\\ & \;\;+\lambda \sum_{r=1}^{\max_{i}r_{i}}\sum_{i=1}^{m}\text{tr}(G_{i}^{\text{T}}\Theta_{i}G_{i})\\ \text{s.t.} & G_{i}\geq O,e^{T}G_{i}e-s_{\left(k\right)}(\text{vec}(G_{i}))=0,\\ \; & i=1,\ldots ,m, \end{array}\right.$$ (2)

Instead of Eq (2), we consider the following penalized problem:

$$\min_{\begin{array}{c} S_{ij}\in {\mathbb{R}}^{k_{i}\times k_{j}},1\leq i,j\leq m\\ G_{i}\in {\mathbb{R}}^{n_{i}\times k_{i}},1\leq i\leq m \end{array}}F(S,G):=f(S,G)+g(G)$$ (3)

where


$$f(S,G)=\sum_{i,j}\omega_{ij}\|R_{ij}-G_{i}S_{ij}G_{j}^{\text{T}}{\|}_{F}^{2}+\lambda \sum_{r=1}^{\max_{i}r_{i}}\sum_{i=1}^{m}\text{tr}(G_{i}^{\text{T}}\Theta_{i}G_{i}),$$

$$g(G)=I_{𝒞}(G)+\lambda_{G}\sum_{i=1}^{m}(e^{T}G_{i}e-s_{\left(k\right)}(\text{vec}(G_{i})))$$

$$:=I_{𝒞}(G)+g_{1}(G)-g_{2}(G),$$

where $\lambda_{G}>0$ is a penalty parameter and $I_{𝒞}$ denotes the indicator function of the set $𝒞=\{G=\text{diag}(G_{1},\ldots ,G_{n})\;|\;G_{i}\in {\mathbb{R}}^{n_{i}\times k_{i}},G_{i}\geq O,i=1,\ldots ,m\}$, i.e., $I_{𝒞}(G)=0$ if G∈𝒞 and $I_{𝒞}(G)=+\infty$, otherwise.

### 2.3 Algorithm for APDCA

**Fig 1:**
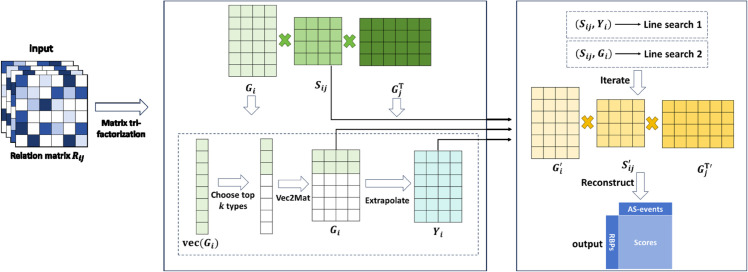
The operating principle of APDCA. APDCA iteratively optimizes the low-rank matrices (*G*_*i*_) of multiple relational data matrices through matrix tri-factorization, and updates (*G*_*i*_) by selecting the top *k* relationships based on sparsity. Then construct an extrapolated point *Y*_*i*_. Then iterate *S*_*ij*_, *Y*_*i*_ by LS-1 if the condition is satisfied, otherwise iterate *S*_*ij*_ and *G*_*i*_ by LS-2. Finally reconstruct the relation matrix and get association scores and rank.

We propose an APDCA algorithm to solve the non-convex optimization problem described in Eq (3), incorporating the ideas originally developed for general nonconvex programming [30] (e.g., sparse optimization [31]). The main process of model is shown in Fig 1.

#### 2.3.1 Initialisation.

For each molecular type *i*, we initialize the nonnegative low-rank basis matrix $G_{i}^{\left(0\right)}\in {\mathbb{R}}^{n_{i}\times k_{i}}$ by performing tri-factorization over the relational matrices *R*_*ij*_ involving the *i*-th object type. Once $G_{i}^{\left(0\right)}$ is obtained, we set $G_{i}^{\left(1\right)}=Z_{i}^{\left(1\right)}=G_{i}^{\left(0\right)}$ to ensure that the first extrapolation step is well-defined. The corresponding interaction matrix *S*^(1)^ is initialized by minimizing the squared Frobenius norm:


$$\min_{S}\|R_{ij}-G_{i}S_{ij}G_{j}^{\text{T}}{\|}_{F}^{2}.$$

Taking the derivative with respect to *S* and setting it to zero yields the closed-form solution:


$$S={\left((G^{\left(1\right)}{)}^{T}G^{\left(1\right)}\right)}^{†}(G^{\left(1\right)}{)}^{T}RG^{\left(1\right)}{\left((G^{\left(1\right)}{)}^{T}G^{\left(1\right)}\right)}^{†}.$$

We record the initial objective $c_{1}=F(S^{\left(1\right)},G^{\left(1\right)})$, set the FISTA parameter $\theta^{\left(1\right)}=1$ and the non-monotone weight *q*_1_ = 1. The Armijo decrease threshold δ>0, the history decay factor τ∈(0,1). We list the initialization of all variables in Table 1.

**Table 1:** Initialization of variables in the APDCA algorithm.

Variable	Initialization Description
$Z_{i}^{\left(1\right)}$	$Z_{i}^{\left(1\right)}=G_{i}^{\left(0\right)}=G_{i}^{\left(1\right)}\geq 0$ for i=1,…,m
$G_{i}^{\left(0\right)},G_{i}^{\left(1\right)}$	≥0
$\omega_{ij}$	$\omega_{ij}>\;0$ for i,j=1,…,m
*λ*	> 0
$\lambda_{G}$	> 0
$\theta^{\left(0\right)}$	0
$\theta^{\left(1\right)}$	1
*δ*	> 0
*τ*	0<τ<1
*q*_1_	1
*c*_1_	*F*(*S*^(1)^,*G*^(1)^)
*S* ^(1)^	${\left((G^{\left(1\right)}{)}^{T}G^{\left(1\right)}\right)}^{†}(G^{\left(1\right)}{)}^{T}RG^{\left(1\right)}{\left((G^{\left(1\right)}{)}^{T}G^{\left(1\right)}\right)}^{†}$
*S* ^(0)^	$S^{\left(0\right)}=S^{\left(1\right)}$
$\omega_{ij}^{\left(1\right)}$	$\frac{1}{2\|R_{ij}-G_{i}^{\left(1\right)}S_{ij}^{\left(1\right)}{\left(G_{j}^{\left(1\right)}\right)}^{T}{\|}_{F}^{2}}$
$l_{1},l_{2}$	> 0
*η*	> 1
ℓ	1

#### 2.3.2 Extrapolation.

At iteration ℓ, we construct an extrapolated point $Y^{\left(\ell \right)}$ by

$$Y^{\left(\ell \right)}=G^{\left(\ell \right)}+\frac{\theta^{\left(\ell -1\right)}}{\theta^{\left(\ell \right)}}\left(Z^{\left(\ell \right)}-G^{\left(\ell \right)}\right)+\frac{\theta^{\left(\ell -1\right)}-1}{\theta^{\left(\ell \right)}}\left(G^{\left(\ell \right)}-G^{\left(\ell -1\right)}\right),$$ (4)

The step size pair $\left(l_{1},l_{2}\right)$ used in the gradient and proximal updates is initialized at this stage. Rather than using fixed values, *l*_1_ and *l*_2_ are dynamically computed based on the iterates and gradients to better reflect local curvature and improve convergence. Specifically, *l*_1_ is calculated by:

$$l_{1}=\frac{\left\langle S^{\left(\ell \right)}-S^{\left(\ell -1\right)},S^{\left(\ell \right)}-S^{\left(\ell -1\right)}\right\rangle }{\left\langle S^{\left(\ell \right)}-S^{\left(\ell -1\right)},\nabla_{S}f(S^{\left(\ell \right)},Y^{\left(\ell \right)})-\nabla_{S}f(S^{\left(\ell -1\right)},Y^{\left(\ell \right)})\right\rangle },$$ (5a)

and *l*_2_ is similarly computed from the *G*-update as:

$$l_{2}=\frac{\left\langle Y^{\left(\ell \right)}-G^{\left(\ell -1\right)},Y^{\left(\ell \right)}-G^{\left(\ell -1\right)}\right\rangle }{\left\langle Y^{\left(\ell \right)}-G^{\left(\ell -1\right)},\nabla_{G}f(S^{\left(\ell \right)},Y^{\left(\ell \right)})-\nabla_{G}f(S^{\left(\ell \right)},G^{\left(\ell -1\right)})\right\rangle }.$$ (5b)

#### 2.3.3 Line Search Algorithm 1 (LS-1).

With the extrapolated point $Y^{\left(\ell \right)}$ fixed, APDCA performs a forward–backward update to generate the candidate pair $\left(S^{\left(\ell +1\right)},Z^{\left(\ell +1\right)}\right)$. The update for *S* is a standard gradient descent step with respect to the smooth part of the objective:

$$S^{\left(\ell +1\right)}=S^{\left(\ell \right)}-\frac{1}{l_{1}}\nabla_{S}f\left(S^{\left(\ell \right)},Y^{\left(\ell \right)}\right),$$ (6)

For the *Z* update, we perform a proximal step as:

$$Z^{\left(\ell +1\right)}={prox}_{(g_{1}+I_{𝒞})/l_{2}}\left(Y^{\left(\ell \right)}-\frac{1}{l_{2}}\nabla_{G}f(S^{\left(\ell \right)},Y^{\left(\ell \right)})+\frac{1}{l_{2}}W(Y^{\left(\ell \right)})\right),$$ (7)

where $W(Y^{\left(\ell \right)})$ is a subgradient of *g*_2_ at $Y^{\left(\ell \right)}$, and


$${prox}_{(g_{1}+I_{𝒞})/l_{2}}(X):=\arg\min_{Z}\left(\frac{1}{l_{2}}(g_{1}(Z)+I_{𝒞}(Z))+\frac{1}{2}\|Z-X{\|}_{F}^{2}\right).$$

The proximal operator handles the nonsmooth components, including the sparsity constraint and nonnegativity projection. Once $\left(S^{\left(\ell +1\right)},Z^{\left(\ell +1\right)}\right)$ is obtained, LS-1 evaluates whether it provides sufficient descent according to an Armijo-type condition:

$$F(S^{\left(\ell +1\right)},Z^{\left(\ell +1\right)})\leq c_{\ell }-\delta (\|S^{\left(\ell +1\right)}-S^{\left(\ell \right)}{\|}_{F}^{2})+\|Z^{\left(\ell +1\right)}-Y^{\left(\ell \right)}{\|}_{F}^{2})$$ (8)

If this inequality is satisfied, then the extrapolated update is accepted by setting $G^{\left(\ell +1\right)}=Z^{\left(\ell +1\right)}$, and the algorithm proceeds to the next iteration via the fast track.

#### 2.3.4 Line Search Algorithm 2 (LS-2).

When the condition in Eq (8) is repeatedly violated even after reducing the extrapolation weight, the algorithm switches to a secondary back-tracking scheme without extrapolation. In this case, the update is recomputed directly from the current iterate $G^{\left(\ell \right)}$, using an alternative gradient-proximal update.

The step sizes *l*_1_ and *l*_2_ are first estimated by:

$$l_{1}=\frac{\left\langle S^{\left(\ell \right)}-S^{\left(\ell -1\right)},S^{\left(\ell \right)}-S^{\left(\ell -1\right)}\right\rangle }{\left\langle S^{\left(\ell \right)}-S^{\left(\ell -1\right)},\nabla_{S}f(S^{\left(\ell \right)},G^{\left(\ell \right)})-\nabla_{S}f(S^{\left(\ell -1\right)},G^{\left(\ell \right)})\right\rangle },$$ (9a)

$$l_{2}=\frac{\left\langle G^{\left(\ell \right)}-G^{\left(\ell -1\right)},G^{\left(\ell \right)}-G^{\left(\ell -1\right)}\right\rangle }{\left\langle G^{\left(\ell \right)}-G^{\left(\ell -1\right)},\nabla_{G}f(S^{\left(\ell \right)},G^{\left(\ell \right)})-\nabla_{G}f(S^{\left(\ell \right)},G^{\left(\ell -1\right)})\right\rangle }$$ (9b)

With these step sizes, the new candidate pair $\left(S^{\left(\ell +1\right)},V^{\left(\ell +1\right)}\right)$ is computed as:

$$S^{\left(\ell +1\right)}=S^{\left(\ell \right)}-\frac{1}{l_{1}}\nabla_{S}f(S^{\left(\ell \right)},G^{\left(\ell \right)}),$$ (10)

$$V^{\left(\ell +1\right)}={prox}_{(g_{1}+I_{𝒞})/l_{2}}\left(G^{\left(\ell \right)}-\frac{1}{l_{2}}\nabla_{G}f(S^{\left(\ell \right)},G^{\left(\ell \right)})+\frac{1}{l_{2}}W(G^{\left(\ell \right)})\right)$$ (11)

The proximal operator handles the nonsmooth sparsity and nonnegativity constraints, as in Eq (7). LS-2 then checks the same Armijo-type descent condition:

$$F(S^{\left(\ell +1\right)},V^{\left(\ell +1\right)})\leq c_{\ell }-\delta (\|S^{\left(\ell +1\right)}-S^{\left(\ell \right)}{\|}_{F}^{2})+\|V^{\left(\ell +1\right)}-G^{\left(\ell \right)}{\|}_{F}^{2})$$ (12)

If this condition is not satisfied, the step sizes *l*_1_ and *l*_2_ are scaled by a factor η>1 and clipped within prescribed bounds:

$$l_{1}\leftarrow \min\left\{\max\{l_{\min}^{1},\eta l_{1}\},\;l_{\max}^{1}\right\},\;l_{2}\leftarrow \min\left\{\max\{l_{\min}^{2},\eta l_{2}\},\;l_{\max}^{2}\right\},$$ (13)

and the updates in Eqs (10)–(11) are recomputed accordingly. The process is repeated until the condition in Eq (12) is met, ensuring convergence even when extrapolation fails.

#### 2.3.5 Final updates and convergence.

After each iteration, the algorithm updates the weights $\omega_{ij}^{\left(\ell +1\right)}$ based on the reconstruction error to down-weight unreliable data sources, and refreshes the FISTA parameter $\theta^{\left(\ell +1\right)}$ along with the non-monotone envelope $c_{\ell +1}$.

**Algorithm 1 APDCA** Accelerated Proximal DC Algorithm.

1: Initialize $G_{i}^{0},G_{i}^{1},S_{i}^{1},Z_{i}^{1}$

2: Compute *Y* by Eq (4)

3: Compute $\left(S^{\left(\ell +1\right)},Z^{\left(\ell +1\right)}\right)$ by LS-1.

4: **If** Eq (8) is satisfied **then**


$$G^{\left(\ell +1\right)}=Z^{\left(\ell +1\right)}.$$

**Else** Compute $\left(S^{\left(\ell +1\right)},V^{\left(\ell +1\right)}\right)$ by LS-2 and set


$$(S^{\left(\ell +1\right)},G^{\left(\ell +1\right)})=\left\{ \begin{array}{cc} (S^{\left(\ell +1\right)},Z^{\left(\ell +1\right)}),\text{ if } & F(S^{\left(\ell +1\right)},Z^{\left(\ell +1\right)})\\ & \leq F(S^{\left(\ell +1\right)},V^{\left(\ell +1\right)}),\\ (S^{\left(\ell +1\right)},V^{\left(\ell +1\right)}), & \text{otherwise}. \end{array}\right.$$

**End (If)**

5: $\omega_{ij}^{\left(\ell +1\right)}=1/(2\|R_{ij}-G_{i}^{\left(\ell +1\right)}S_{ij}^{\left(\ell +1\right)}{\left(G_{j}^{\left(\ell +1\right)}\right)}^{T}{\|}_{F}^{2})$

6: $\theta^{\left(\ell +1\right)}=\frac{\sqrt{4{\left(\theta^{\left(\ell \right)}\right)}^{2}+1}+1}{2}.$

7: $q_{\ell +1}=\tau q_{\ell }+1.$

8: $c_{\ell +1}=\frac{\tau q_{\ell }c_{\ell }+F(S^{\left(\ell +1\right)},G^{\left(\ell +1\right)})}{q_{\ell +1}}.$

9: Replace ℓ by ℓ+1 and go to step 2.

**Algorithm 2 LS-1 algorithm** (Compute $\left(S^{\left(\ell +1\right)},Z^{\left(\ell +1\right)}\right)$ with line search).

1: Initialize *l*_1_, *l*_2_, *η*

2: Update $l_{1},l_{2}$ by Eq (5a) and Eq (5b)

3: Compute $S^{\left(\ell +1\right)}$ by Eq (6) and $Z^{\left(\ell +1\right)}$ by Eq (7)

**Repeat** until Eq (8) is satisfied.

Replace *l*_1_, *l*_2_ by Eq (13), compute $S^{\left(\ell +1\right)}$, $Z^{\left(\ell +1\right)}$

**end (Repeat)**

4: $l_{1}^{\left(\ell \right)}=l_{1}$ and $l_{2}^{\left(\ell \right)}=l_{2}$.

**Algorithm 3 LS-2 algorithm** (Compute $\left(S^{\left(\ell +1\right)},V^{\left(\ell +1\right)}\right)$ with line search).

1: Initialize *l*_1_, *l*_2_, *η*

2: Update $l_{1},l_{2}$ by Eq (9a) and Eq (9b)

3: Compute $S^{\left(\ell +1\right)}$ by Eq (10) and $V^{\left(\ell +1\right)}$ by Eq (11)

**Repeat** until Eq (12) is satisfied.

Replace *l*_1_, *l*_2_ by Eq (13), compute $S^{\left(\ell +1\right)}$, $V^{\left(\ell +1\right)}$

**end (Repeat)**

4: $l_{1}^{\left(\ell \right)}=l_{1}$ and $l_{2}^{\left(\ell \right)}=l_{2}$.

### 2.4 Convergence proof

In this section we introduce the essential logic of convergence proof for the algorithm above, the detailed version see S1 Text.

First, the back-tracking criteria embedded in LS-1 and LS-2 create a descent envelope $c_{\ell }$ that is quasi-Fejér monotone: every accepted update decreases $c_{\ell }$ by at least a multiple of the squared iterate gap. Second, because the smooth term *f* is coercive while the regularization *g*_1_ and the non-negativity indicator *I*_*C*_ confine $G^{\left(\ell \right)}$ to a closed cone, all iterates reside in a compact level set of *F*.

With boundedness secured, closedness of the subdifferential mapping $G\;\mapsto \;\partial (g_{1}+I_{C})(G)$ permits passage to the limit in the optimality condition of each proximal step. Hence every accumulation point $\left(S^{⋆},G^{⋆}\right)$ obeys the stationarity condition $0\in \partial F(S^{⋆},G^{⋆})$. Whether the accepted updates come from LS-1 (on extrapolated points) or LS-2 (on current points) is immaterial. The argument treats the two cases symmetrically and covers all subsequences.

If the objective *F* moreover satisfies the Kurdyka–Łojasiewicz (KL) inequality—which is automatic for the semi-algebraic penalties used in Section 1—the standard DC-programming framework further yields full sequence convergence together with an explicit sub-linear rate.

## 3 Results and discussions

### 3.1 Experimental setup

**Fig 2:**
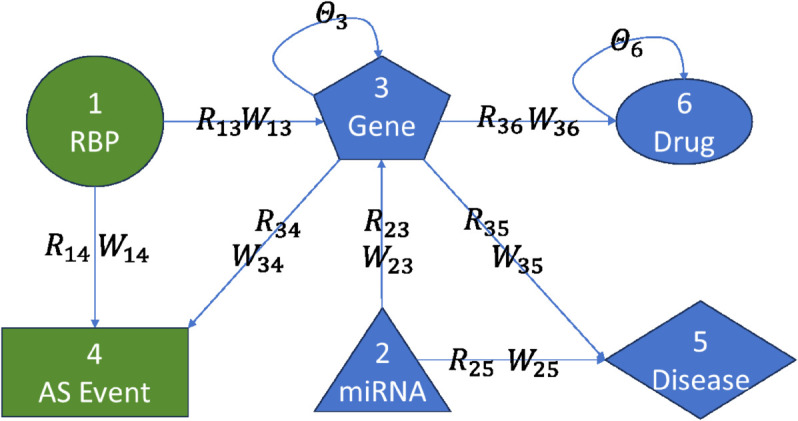
Schematic illustration of the multi-type biological network used in APDCA. Six types of biological entities are represented: RNA-binding proteins (RBPs), miRNAs, genes, alternative splicing (AS) events, diseases, and drugs. Edges between entities denote observed associations *R*_*ij*_ with corresponding weights *W*_*ij*_. Self-loop parameters $\theta_{3}$ and $\theta_{6}$ are used for gene and drug regularization, respectively.

To investigate the performance of APDCA, we consider six core biological object types: RBPs (Type 1), miRNAs (Type 2), genes (Type 3), AS events (Type 4), diseases (Type 5), and drugs (Type 6). We integrated nine distinct relational data sources from public biological databases, covering both inter-relational and intra-relational associations. The relation between different entities is shown in Fig 2.

For RBP-related associations, we collected gene expression data of 1,532 RBPs from a recent census on BRCA samples [32,33] and obtained AS event features by integrating junction reads from The Cancer Genome Atlas with the Mixture-of-Isoforms database [19,34,35]. Associations between RBPs and AS events were established based on Pearson correlation analysis, with more detailed preprocessing procedures provided in S2 Text. This yielded three binary matrices describing relationships among RBPs, genes, and AS events: RBP-genes (*R*_13_), RBP–AS events (*R*_14_) and gene–AS events (*R*_34_).

In addition, we incorporated miRNA–gene and miRNA–disease associations from miRTarBase [36] and HMDD [37] respectively; gene–disease and gene–drug associations from DisGeNET [38] and DrugBank [39]; and gene–gene and drug–drug interactions from BioGrid [40] and DrugBank [39]. All datasets were retrieved from the latest versions of these databases.

We perform 5-fold cross-validation on the RBP–AS event association matrix (*R*_14_). In each fold, the known associations were partitioned into training and testing sets along the row dimension, while all other relational matrices remained unchanged throughout the evaluation.

### 3.2 Performance comparison with existing methods

**Fig 3:**
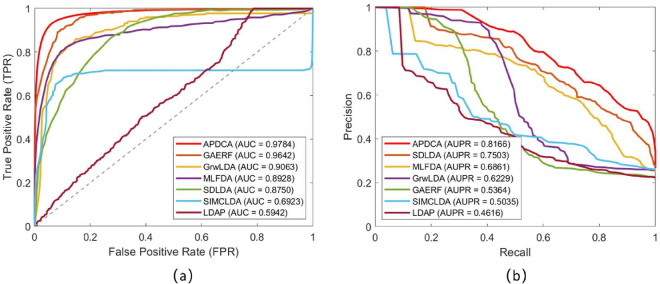
Comparison of predictive performance under 5-fold cross-validation. (a) ROC curves with AUC values. (b) Precision-Recall curves with AUPR values. APDCA achieves the highest scores across both metrics.

To evaluate the predictive performance of APDCA, we conduct experiments on benchmark datasets using 5-fold cross-validation, and compared it against six representative models: SDLDA [16], LDAP [17], GAERF [18], MLFDA [20], SIMCLDA [21], and GrwLDA [22]. Performance is assessed using two widely adopted metrics—AUC (area under the ROC curve) and AUPR (area under the Precision-Recall curve). As shown in Fig 3, APDCA consistently achieves the best results across both metrics. This reflects its ability to integrate heterogeneous biological relationships in a unified optimization framework while adaptively filtering noise via sparse constraints and low-rank factorization. Such a structure enhances both discriminative capability and generalization under sparse label scenarios.

In terms of AUC performance, APDCA obtains the highest score of 0.9784, outperforming all baselines. The ROC curve in Fig 3(a) shows that the closest result comes from GAERF (0.9642), followed by GrwLDA (0.9063) and MLFDA (0.8928). In contrast, SIMCLDA and LDAP exhibit relatively poor performance, with AUCs of 0.6923 and 0.5942, respectively. The strong AUC of GAERF can be attributed to its graph-enhanced learning structure, but its reliance on static graph construction may limit adaptability across data types. GrwLDA, based on topic-aware random walks, captures global associations but lacks precision in local structure modeling. SIMCLDA, despite using inductive matrix completion, is constrained by Gaussian kernel similarity and fixed feature propagation, making it less effective in multi-relational tasks. In contrast, APDCA dynamically learns embedding spaces with joint low-rank representations, enabling more expressive modeling across different biological object types.

The AUPR comparison further highlights APDCA’s robustness under label imbalance. As shown in the Precision-Recall curve in Fig 3(b), APDCA achieves an AUPR of 0.8166, significantly higher than SDLDA (0.7503), MLFDA (0.6861), and GrwLDA (0.6229). GAERF, despite its strong AUC, drops sharply to 0.5364 in AUPR, indicating a large number of false positives. LDAP and SIMCLDA also yield low AUPRs of 0.4616 and 0.5035, respectively, likely due to their limited capacity to prioritize true positives under sparse supervision. The weak AUPR of SDLDA suggests sensitivity to hyperparameter tuning and difficulty in handling noisy unlabeled samples. In contrast, APDCA’s proximal DC framework and envelope update mechanism promote stable convergence and strong discriminative structure, allowing it to maintain high precision and recall even in highly imbalanced settings.

### 3.3 Algorithm convergence speed

**Fig 4:**
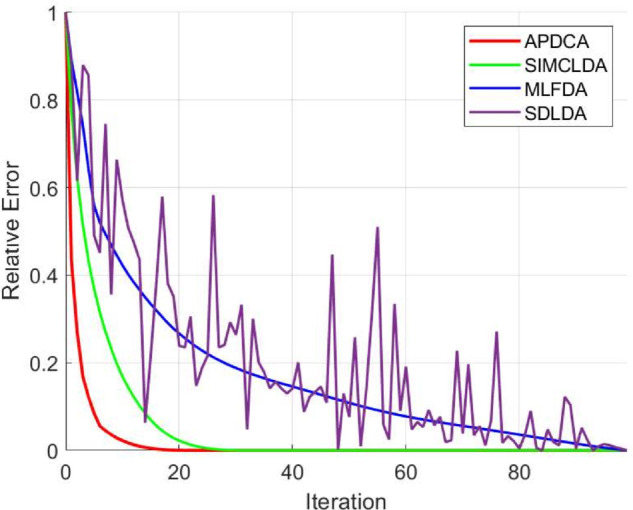
Convergence comparison of APDCA, SIMCLDA, MLFDA, and SDLDA over 100 iterations using relative error as the evaluation metric.

We further assess the convergence speed of APDCA by comparing it with SIMCLDA, MLFDA, and SDLDA to show its accelerated convergence. The number of iterations is set to 100 for all methods. To evaluate convergence, we use relative error computed via max–min normalization. As shown in Fig 4, APDCA demonstrates the fastest convergence, with its relative error dropping below 0.05 within the first 10 iterations and reaching zero around the 20th iteration. SIMCLDA also shows a steady convergence trend, although at a much slower rate. In contrast, the convergence curve of SDLDA is unstable and exhibits noticeable oscillations, making it less reliable. MLFDA converges more smoothly than SDLDA but still requires more iterations than APDCA. These results suggest that APDCA not only achieves convergence more quickly but also does so more consistently.

### 3.4 Case studies of APDCA

**Fig 5:**
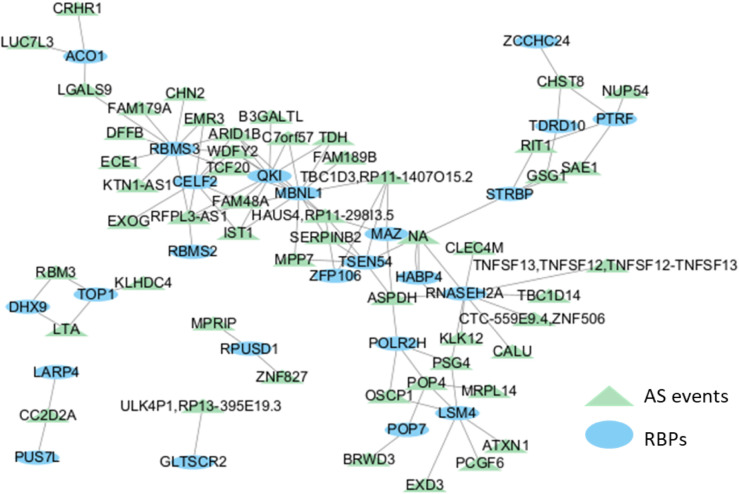
Network showing the RBP–AS event associations during EMT.

To assess the biological validity of the predicted associations from APDCA, We report the top 100 pairs of RBPsand AS events associations during EMT which are illustrated by Fig 5. Fig 5 shows that many RBPs form groups to regulate AS events. We further conducted a case study on the RNA-binding protein QKI, which has been implicated in regulating alternative splicing during epithelial–mesenchymal transition (EMT) and cancer progression [9]. We extracted the top 10 AS events most strongly associated with QKI as predicted by our model. The OncoSplicing database was used for evaluation, as it aggregates clinically relevant AS events across 33 human cancers, many of which are closely related to EMT progression, and provides regulatory support including eCLIP peaks, motif mappings, and co-expression data [26].

Among the top 10 predicted QKI–AS event pairs shown in Table 2, 7 out of 10 were supported by at least one type of regulatory evidence in OncoSplicing. Specifically, 6 AS events showed eCLIP peak support, such as IST1 with 9 peaks and ARID1B with 5 peaks, and 5 events exhibited significant co-expression with QKI in cancer samples, such as IST1 with correlations observed in 5 cancer types. In terms of regulatory confidence, 2 AS events IST1 and ARID1B were labeled as high confidence (high rate), while several others were labeled mild based on the integrated evidence. Only three events AS1, FAM48A, and TDH had no evidence recorded in the current database release. As a representative example, the association between QKI and the exon skipping event in IST1 is supported by 9 eCLIP peaks and correlated expression in 5 distinct TCGA cancer types. This multi-modal support strongly indicates a regulatory role of QKI over this AS event, consistent with prior biological studies. Overall, the validation results suggest that APDCA is capable of prioritizing biologically meaningful RBP AS pairs.

**Table 2:** Top 10 QKI–AS Event Associations Predicted by APDCA and Their Evidence in OncoSplicing.

AS Event	Peaks	Corr Cancer	Rate
IST1	9	5	High
AS1	–	–	–
ARID1B	5	3	High
TCF20	3	2	Mild
HAUS4	4	1	Mild
WDFY2	1	0	Mild
FAM48A	–	–	–
TDH	–	–	–
C7orf57	0	0	Mild
B3GALTL	1	0	Mild

We also evaluated APDCA on an independent lncRNA–disease dataset to further assess its generalizability, and the detailed results are provided in S3 Text.

### 3.5 Parameter analysis

To assess the robustness and stability of APDCA, we performed parameter sensitivity analyses on the regularization parameters *λ* and $\lambda_{G}$, as well as the sparsity control parameter *k*. We aim to evaluate how different settings affect model performance, measured by AUC.

#### 3.5.1 Effect of *λ* and $\lambda_{G}$.

**Fig 6:**
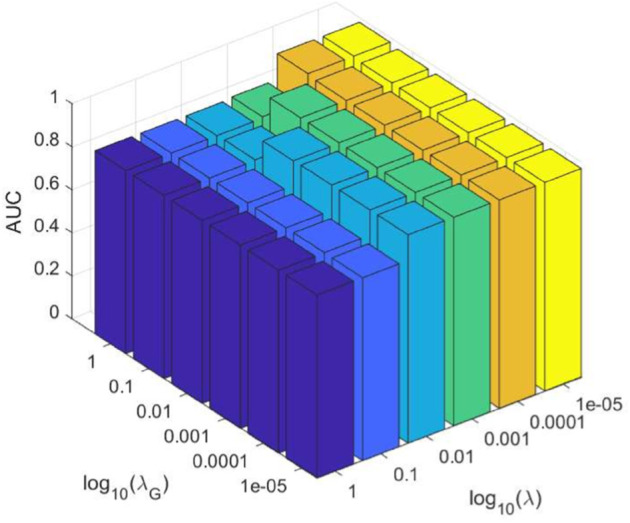
AUC surface with respect to regularization parameters *λ* and $\lambda_{G}$. Performance improves as both parameters decrease and stabilizes at low values.

We first explored the impact of the regularization parameters *λ* and $\lambda_{G}$ with 5-fold cross-validation, which control the low-rank representation and sparsity constraints, respectively. A grid search is performed over $\lambda ,\lambda_{G}\in 1,10^{-1},10^{-2},10^{-3},10^{-4},10^{-5}$, and the AUC values for each parameter pair are summarized in Fig 6.

As shown in Table 3, the highest AUC value of 0.9784 is achieved when λ=0.0001 and $\lambda_{G}=1$. And when *λ* is within the range of 10^−2^,10^−3^,10^−4^,10^−5^ and $\lambda_{G}$ is within the range of 10^−2^,10^−3^,10^−4^,10^−5^, the model consistently obtains high AUC values above 0.969. In contrast, when both parameters are set above 0.01, the AUC drops to around 0.85, indicating a clear degradation in performance. Based on these results, we recommend choosing $\lambda \in 10^{-2},10^{-3},10^{-4},10^{-5}$ and $\lambda_{G}\in 10^{-2},10^{-3},10^{-4},10^{-5}$ to ensure strong and stable model performance.

**Table 3:** AUC values under different combinations of regularization parameters *λ* and $\lambda_{G}$.

$\lambda_{G}\backslash \lambda$	1	10^−1^	10^−2^	10^−3^	10^−4^	10^−5^
1	0.8523	0.8520	0.8518	0.8586	**0.9784**	0.9784
10^−1^	0.8521	0.8518	0.8554	0.9695	0.9695	0.9695
10^−2^	0.8517	0.8516	0.9666	0.9695	0.9694	0.9695
10^−3^	0.8521	0.8514	0.9694	0.9694	0.9694	0.9694
10^−4^	0.8519	0.8521	0.9695	0.9694	0.9695	0.9694
10^−5^	0.8521	0.8514	0.9694	0.9695	0.9695	0.9694

#### 3.5.2 Effect of *k.*

**Fig 7:**
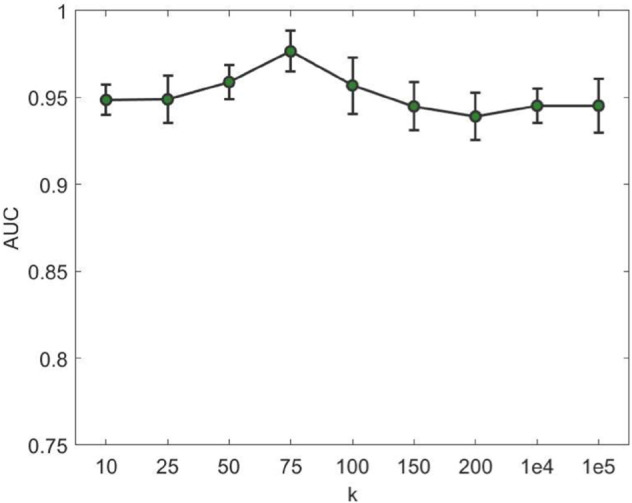
AUC sensitivity of APDCA to the sparse control parameter *k* under 5-fold cross-validation. Performance peaks at *k* = 75 and remains stable across a wide range.

We conducted a sensitivity analysis on the sparse control parameter *k*, which regulates the sparsity level of the reconstructed relationship matrix in our APDCA framework. As shown in Fig 7, we varied *k* over a wide range from 10 to 10^5^ and evaluated the model performance using the AUC metric under 5-fold cross-validation.

The results demonstrate that the performance of APDCA remains robust across a broad range of *k* values. Specifically, AUC values consistently exceed 0.94 for all settings, with the highest performance observed around *k* = 75. This suggests that moderate sparsity levels provide an optimal balance between noise suppression and information preservation in the reconstructed association matrix. When k is too small, important associations may be missed, while excessively large *k* may introduce redundant or noisy connections, slightly degrading performance.

Overall, this analysis confirms that APDCA is not overly sensitive to the choice of *k*, and a reasonably chosen *k* (e.g., 50–100) yields stable and high-quality predictions.

## 4 Conclusions

Identifying regulatory associations between RNA-binding proteins (RBPs) and alternative splicing (AS) events during epithelial–mesenchymal transition (EMT) is essential for understanding cell state transitions and cancer metastasis. However, experimental validation remains costly and context-dependent. To address this, we proposed APDCA (Accelerated Proximal DC Algorithm), a novel computational method designed to predict RBP–AS associations during EMT. APDCA integrates heterogeneous biological data using a sparse low-rank matrix factorization framework. A sparsity constraint is applied to reflect the selective nature of RBP–AS regulation. The resulting non-convex problem is reformulated via Difference-of-Convex (DC) programming and solved using a proximal optimization scheme with Nesterov-accelerated updates, which improves both convergence speed and stability. Experimental results show that APDCA outperforms six baseline models in AUC and AUPR across 5-fold cross-validation. A case study involving the EMT-related RBP QKI demonstrates the biological relevance of the predicted associations, supported by external evidence from the OncoSplicing database. While APDCA achieves strong predictive performance, its effectiveness depends on the quality and completeness of input data. Beyond this specific application, APDCA offers a generalizable optimization framework that can be readily extended to other molecular association prediction tasks, such as lncRNA–disease or miRNA–disease relationships. Future work will incorporate dynamic transcriptomic datasets and extend the model to additional types of AS events. Moreover, integrating APDCA with deep learning architectures or probabilistic inference schemes could further enhance its scalability and interpretability, broadening its impact across multi-omics integration and systems biology research.

## Supporting information

S1 Text Convergence proof of APDCA. (PDF)

S2 Text Data preprocessing procedures. (PDF)

S3 Text Evaluation of APDCA on lncRNA–disease prediction. (PDF)
