# Supplementary material for: APDCA: An accurate and effective method for predicting associations between RBPs and AS-events during epithelial-mesenchymal transition
Source: PLoS Comput Biol. 2025 Nov 6;21(11):e1013665. doi: 10.1371/journal.pcbi.1013665 (PMC12604773; doi:10.1371/journal.pcbi.1013665)
Supplement: S1 Text — (PDF) [file pcbi.1013665.s001.pdf]

Convergence Proof

Algorithm 1 APDCA Accelerated Proximal DC Algorithm

1: Choose $Z_i^{(1)} = G_i^{(0)} = G_i^{(1)} \geq 0$ for $i = 1, \ldots m$, $\omega_{ij} > 0$ for $i, j = 1, \ldots m$, $\lambda > 0$, $\lambda_G > 0$, $\theta^{(0)} = 0$, $\theta^{(1)} = 1$, $\delta > 0$, $0 < \tau < 1$, $q_1 = 1$, $c_1 = F(S^{(1)}, G^{(1)})$ with $S^{(1)} = ((G^{(1)})^T G^{(1)})^\dagger (G^{(1)})^T R G^{(1)} ((G^{(1)})^T G^{(1)})^\dagger = S^{(0)}$, and $\omega_{ij}^{(1)} = 1/(2\|R_{ij} - G_i^{(1)} S_{ij}^{(1)} (G_j^{(1)})^T\|_F^2)$ for $i, j = 1, \ldots, m$. Let $\ell := 1$.

2: Compute

$$Y^{(\ell)} = G^{(\ell)} + \frac{\theta^{(\ell-1)}}{\theta^{(\ell)}} \left(Z^{(\ell)} - G^{(\ell)} \right) + \frac{\theta^{(\ell-1)} - 1}{\theta^{(\ell)}} \left(G^{(\ell)} - G^{(\ell-1)} \right)$$

and compute $(S^{(\ell+1)}, Z^{(\ell+1)})$ by LS-1.

If $F(S^{(\ell+1)}, Z^{(\ell+1)}) \leq c_\ell - \delta(\|S^{(\ell+1)} - S^{(\ell)}\|_F^2 + \|Z^{(\ell+1)} - Y^{(\ell)}\|_F^2)$ **then**

$$G^{(\ell+1)} = Z^{(\ell+1)}. \tag{1}$$

else

Compute $(S^{(\ell+1)}, V^{(\ell+1)})$ by LS-2 and set

$$(S^{(\ell+1)}, G^{(\ell+1)}) = \begin{cases} (S^{(\ell+1)}, Z^{(\ell+1)}), & \text{if} \quad F(S^{(\ell+1)}, Z^{(\ell+1)}) \\ & \quad \leq F(S^{(\ell+1)}, V^{(\ell+1)}), \\ (S^{(\ell+1)}, V^{(\ell+1)}), & \text{otherwise.} \end{cases} \tag{2}$$

end (If)

3: $\omega_{ij}^{(\ell+1)} = 1/(2\|R_{ij} - G_i^{(\ell+1)} S_{ij}^{(\ell+1)} (G_j^{(\ell+1)})^T\|_F^2)$ for $i, j = 1, \ldots, m$.

4: $\theta^{(\ell+1)} = \dfrac{\sqrt{4(\theta^{(\ell)})^2 + 1} + 1}{2}$.

5: $q_{\ell+1} = \tau q_\ell + 1$.

6: $c_{\ell+1} = \dfrac{\tau q_\ell c_\ell + F(S^{(\ell+1)}, G^{(\ell+1)})}{q_{\ell+1}}$.

7: Replace ℓ by $\ell + 1$ and go to step 2.

One may establish the convergence of Algorithm 1 as in [1, Theorem 5]. However, it assume that f is continuous differentiable with Lipschitz continuous gradient, g_1 is lower semicontinuous and convex, g_2 is continuous and convex, and F is bounded from below and coercive. This is not true for our problem. Therefore, we need some other assumption to guarantee the convergence of Algorithm 1.

To show the convergence of Algorithm 1, we need the following proposition (see for instance [3, 4]).

Proposition 0.1. *Let $\{G^{(\ell)}\}$ and $\{U^{(\ell)}\}$ be two sequence such that $G^{(\ell)} \to G^*$, $U^{(\ell)} \to U^*$, $g_1(G^{(\ell)}) + I_\mathcal{C}(G^{(\ell)}) \to g_1(G^*) + I_\mathcal{C}(G^*)$, and $U^{(\ell)} \in \partial g_1(G^{(\ell)})$. Then one has $U^* \in \partial g_1(G^*) + I_\mathcal{C}(G^*)$.*

Under some mild assumptions, we have the following result on the global convergence of Algorithm 1. The proof is similar to that of [2, Theorem 4] or [1, Theorem 5]. Here, we give the proof since different assumptions are involved.

Algorithm 2 LS-1 algorithm (Compute $(S^{(\ell+1)}, Z^{(\ell+1)})$ with line search)

1: Choose $0 < l^1_{\min} < l^1_{\max}$, $0 < l^2_{\min} < l^2_{\max}$, and $\eta > 1$.

2: Take

$$l_1 = \frac{\langle S^{(\ell)} - S^{(\ell-1)}, S^{(\ell)} - S^{(\ell-1)} \rangle}{\langle S^{(\ell)} - S^{(\ell-1)}, \nabla_S f(S^{(\ell)}, Y^{(\ell)}) - \nabla_S f(S^{(\ell-1)}, Y^{(\ell)}) \rangle},$$

$$l_2 = \frac{\langle Y^{(\ell)} - G^{(\ell-1)}, Y^{(\ell)} - G^{(\ell-1)} \rangle}{\langle Y^{(\ell)} - G^{(\ell-1)}, \nabla_G f(S^{(\ell)}, Y^{(\ell)}) - \nabla_G f(S^{(\ell)}, G^{(\ell-1)}) \rangle}$$

and compute

$$S^{(\ell+1)} = S^{(\ell)} - \frac{1}{l_1} \nabla_S f(S^{(\ell)}, Y^{(\ell)}),$$

$$Z^{(\ell+1)} = \mathbf{prox}_{(g_1+I_{\mathcal{C}})/l_2}\Big(Y^{(\ell)} - \frac{1}{l_2}\nabla_G f(S^{(\ell)}, Y^{(\ell)}) + \frac{1}{l_2} W(Y^{(\ell)})\Big).$$

where $W(Y^{(\ell)})$ is a subgradient of g_2 at $Y^{(\ell)}$, and

$$\mathbf{prox}_{(g_1+I_{\mathcal{C}})/l_2}(X) := \arg\min_Z \left(\frac{1}{l_2}(g_1(Z) + I_{\mathcal{C}}(Z)) + \frac{1}{2}\|Z - X\|_F^2 \right)$$

Repeat until $F(S^{(\ell+1)}, Z^{(\ell+1)}) \le c_\ell - \delta(\|S^{(\ell+1)} - S^{(\ell)}\|_F^2) + \|Z^{(\ell+1)} - Y^{(\ell)}\|_F^2)$.

Replace l_1 by $\min\{\max\{l^1_{\min}, \eta l_1\}, l^1_{\max}\}$, l_2 by $\min\{\max\{l^2_{\min}, \eta l_2\}, l^2_{\max}\}$, and compute

$$S^{(\ell+1)} = S^{(\ell)} - \frac{1}{l_1} \nabla_S f(S^{(\ell)}, Y^{(\ell)}),$$

$$Z^{(\ell+1)} = \mathbf{prox}_{(g_1+I_{\mathcal{C}})/l_2}\Big(Y^{(\ell)} - \frac{1}{l_2}\nabla_G f(S^{(\ell)}, Y^{(\ell)}) + \frac{1}{l_2} W(Y^{(\ell)})\Big).$$

end (Repeat)

3: $l_1^{(\ell)} = l_1$ and $l_2^{(\ell)} = l_2$.

Theorem 0.2. *Let $\{(S^{(\ell)}\}, \{G^{(\ell)}\}, \{Y^{(\ell)}\}, \{V^{(\ell)})\}$ be the sequences generated by Algorithm 1. Let Ω_1 be the set of ℓ at which (1) is satisfied and Ω_2 be the set of ℓ at which (2) is satisfied. Suppose that $\{(S^{(\ell)}\}$ and $\{G^{(\ell)}\}$ are bounded and*

1. *if Ω_1 or Ω_2 is finite, then for any accumulation point (S^*, G^*) of $\{(S^{(\ell)}, G^{(\ell)}\}$, we have $0 \in \partial F(S^*, G^*)$.*

2. *if both Ω_1 and Ω_2 are infinite, then for any accumulation point (S_1^*, Y^*) of $\{(S^{(\ell+1)}, Y^{(\ell)})\}_{\ell \in \Omega_1}$, and any accumulation point (S_2^*, V^*) of $\{(S^{(\ell)}, V^{(\ell+1)})\}_{\ell \in \Omega_2}$, we have $0 \in \partial F(S_1^*, Y^*)$ and $0 \in \partial F(S_2^*, V^*)$.*

Algorithm 3 LS-2 algorithm (Compute $(S^{(\ell+1)}, V^{(\ell+1)})$ with line search)

1: Choose $0 < l^1_{\min} < l^1_{\max}$, $0 < l^2_{\min} < l^2_{\max}$, and $\eta > 1$.

2: Take

$$l_1 = \frac{\langle S^{(\ell)} - S^{(\ell-1)}, S^{(\ell)} - S^{(\ell-1)} \rangle}{\langle S^{(\ell)} - S^{(\ell-1)}, \nabla_S f(S^{(\ell)}, G^{(\ell)}) - \nabla_S f(S^{(\ell-1)}, G^{(\ell)}) \rangle},$$

$$l_2 = \frac{\langle G^{(\ell)} - G^{(\ell-1)}, G^{(\ell)} - G^{(\ell-1)} \rangle}{\langle G^{(\ell)} - G^{(\ell-1)}, \nabla_G f(S^{(\ell)}, G^{(\ell)}) - \nabla_G f(S^{(\ell)}, G^{(\ell-1)}) \rangle}$$

and compute

$$S^{(\ell+1)} = S^{(\ell)} - \frac{1}{l_1} \nabla_S f(S^{(\ell)}, G^{(\ell)}),$$

$$V^{(\ell+1)} = \mathbf{prox}_{(g_1+I_C)/l_2} \Big(G^{(\ell)} - \frac{1}{l_2} \nabla_G f(S^{(\ell)}, G^{(\ell)}) + \frac{1}{l_2} W(G^{(\ell)}) \Big).$$

Repeat until $F(S^{(\ell+1)}, V^{(\ell+1)}) \le c_\ell - \delta(\|S^{(\ell+1)} - S^{(\ell)}\|_F^2) + \|V^{(\ell+1)} - G^{(\ell)}\|_F^2)$.

Replace l_1 by $\min\{\max\{l^1_{\min}, \eta l_1\}, l^1_{\max}\}$, l_2 by $\min\{\max\{l^2_{\min}, \eta l_2\}, l^2_{\max}\}$, and compute

$$S^{(\ell+1)} = S^{(\ell)} - \frac{1}{l_1} \nabla_S f(S^{(\ell)}, G^{(\ell)}),$$

$$V^{(\ell+1)} = \mathbf{prox}_{(g_1+I_C)/l_2} \Big(G^{(\ell)} - \frac{1}{l_2} \nabla_G f(S^{(\ell)}, G^{(\ell)}) + \frac{1}{l_2} W(G^{(\ell)}) \Big).$$

end (Repeat)

3: $l_1^{(\ell)} = l_1$ and $l_2^{(\ell)} = l_2$.

Proof. If the condition $G^{(\ell+1)} = Z^{(\ell+1)}$ is satisfied, then we have

$$\begin{aligned} F(S^{(\ell+1)}, G^{(\ell+1)}) &\le c_\ell - \delta \|S^{(\ell+1)} - S^{(\ell)}\|_F^2 \\ &\qquad - \delta \|G^{(\ell+1)} - Y^{(\ell)}\|_F^2, \\ c_{\ell+1} &= \frac{\tau q_\ell c_\ell + F(S^{(\ell+1)}, G^{(\ell+1)})}{q_{\ell+1}}, \\ &\le \frac{\tau q_\ell c_\ell + c_\ell - \delta \left(\|S^{(\ell+1)} - S^{(\ell)}\|_F^2 + \|G^{(\ell+1)} - Y^{(\ell)}\|_F^2 \right)}{q_{\ell+1}}, \\ &= c_\ell - \frac{\delta \left(\|S^{(\ell+1)} - S^{(\ell)}\|_F^2 + \|G^{(\ell+1)} - Y^{(\ell)}\|_F^2 \right)}{q_{\ell+1}}. \end{aligned}$$

If the condition

$$(S^{(\ell+1)}, G^{(\ell+1)}) = \begin{cases} (S^{(\ell+1)}, Z^{(\ell+1)}), \text{if} & F(S^{(\ell+1)}, Z^{(\ell+1)}) \\ & \le F(S^{(\ell+1)}, V^{(\ell+1)}), \\ (S^{(\ell+1)}, V^{(\ell+1)}), & \text{otherwise} \end{cases}$$

is satisfied, then we have

$$\begin{aligned} F(S^{(\ell+1)}, G^{(\ell+1)}) &\le F(S^{(\ell+1)}, V^{(\ell+1)}) \\ &\le c_\ell - \delta \left(\|S^{(\ell+1)} - S^{(\ell)}\|_F^2 + \|V^{(\ell+1)} - G^{(\ell)}\|_F^2 \right), \\ c_{\ell+1} &\le c_\ell - \frac{\delta (\|S^{(\ell+1)} - S^{(\ell)}\|_F^2 + \|V^{(\ell+1)} - G^{(\ell)}\|_F^2)}{q_{\ell+1}}. \end{aligned}$$

By the definitions of Ω_1 and Ω_2, we have $\Omega_1 \cap \Omega_2 = \emptyset$, $\Omega_1 \cup \Omega_2 = \{1, 2, 3, \cdots\}$, and

$$c_{\ell+1} \leq c_\ell - \frac{\delta(\|S^{(\ell+1)} - S^{(\ell)}\|_F^2 + \|G^{(\ell+1)} - Y^{(\ell)}\|_F^2)}{q_{\ell+1}},$$

for all $\ell \in \Omega_1$ and

$$c_{\ell+1} \leq c_\ell - \frac{\delta(\|S^{(\ell+1)} - S^{(\ell)}\|_F^2 + \|V^{(\ell+1)} - G^{(\ell)}\|_F^2)}{q_{\ell+1}},$$

for all $\ell \in \Omega_2$.

By the definition of q_ℓ, we have

$$q_{\ell+1} = 1 + \sum_{k=1}^{\ell} \tau^k \leq \sum_{k=1}^{\infty} \tau^k = \frac{1}{1-\tau}.$$

Thus,

$$\begin{aligned} \delta(1-\tau)(\|S^{(\ell+1)} - S^{(\ell)}\|_F^2 + \|Z^{(\ell+1)} - Y^{(\ell)}\|_F^2) \leq c_\ell - c_{\ell+1}, \quad \ell \in \Omega_1, \\ \delta(1-\tau)(\|S^{(\ell+1)} - S^{(\ell)}\|_F^2 + \|V^{(\ell+1)} - G^{(\ell)}\|_F^2) \leq c_\ell - c_{\ell+1}, \quad \ell \in \Omega_2. \end{aligned}$$

This yields

$$\begin{aligned} &\sum_{\ell \in \Omega_1} (\|S^{(\ell+1)} - S^{(\ell)}\|_F^2 + \|G^{(\ell+1)} - Y^{(\ell)}\|_F^2) \\ &+ \sum_{\ell \in \Omega_2} (\|S^{(\ell+1)} - S^{(\ell)}\|_F^2 + \|V^{(\ell+1)} - G^{(\ell)}\|_F^2) \\ &\leq \frac{c_1 - c^*}{\delta(1-\tau)} < \infty, \end{aligned}$$

where F^* is the same function value at all accumulation points of F since $F(S^{(\ell+1)}, G^{(\ell+1)}) \leq c_\ell$ for all $\ell \geq 1$. We consider the following three cases.

a) Ω_2 is finite. In this case, there exist $L_0 > 0$ such that $G^{(\ell+1)} = Z^{(\ell+1)}$ for all $\ell > L_0$. Thus,

$$\sum_{\ell=L_0}^{\infty} (\|S^{(\ell+1)} - S^{(\ell)}\|_F^2 + \|G^{(\ell+1)} - Y^{(\ell)}\|_F^2) < \infty,$$

which implies that $\|S^{(\ell+1)} - S^{(\ell)}\|_F \to 0$ and $\|G^{(\ell+1)} - Y^{(\ell)}\|_F \to 0$ as $\ell \to \infty$. By hypothesis, both $\{S^{(\ell)}\}$ and $\{G^{(\ell)}\}$ are bounded. Then we have $\{Y^{(\ell)}\}$ is bounded and thus $\{\nabla f(S^{(\ell)}, Y^{(\ell)})\}$ is bounded. Let S^* and Y^* be arbitrary accumulation point of $\{S^{(\ell)}\}$ and $\{Y^{(\ell)}\}$, respectively. Then it is easy to see that Y^* is an accumulation point of $\{G^{(\ell)}\}$.

From the optimality conditions

$$S^{(\ell+1)} = S^{(\ell)} - \frac{1}{l_1} \nabla_S f(S^{(\ell)}, Y^{(\ell)}), \tag{3}$$

$$Z^{(\ell+1)} = \text{prox}_{(g_1 + I_\mathcal{C})/l_2} \Big(Y^{(\ell)} - \frac{1}{l_2} \nabla_G f(S^{(\ell)}, Y^{(\ell)}) + \frac{1}{l_2} W(Y^{(\ell)}) \Big) \tag{4}$$

and $G^{(\ell+1)} = Z^{(\ell+1)}$, we have

$$\begin{aligned} &0 = \nabla_S f(S^{(\ell)}, Y^{(\ell)}) + l_1^{(\ell)} (S^{(\ell+1)} - S^{(\ell)}), \\ &0 \in \{\nabla_G f(S^{(\ell)}, Y^{(\ell)}) + l_2^{(\ell)} (G^{(\ell+1)} - Y^{(\ell)}) - W(Y^{(\ell)})\} + \partial g_1(G^{(\ell+1)}) + \partial I_\mathcal{C}(G^{(\ell+1)}), \end{aligned}$$

where the second condition is equivalent to

$$-\nabla_G f(S^{(\ell)}, Y^{(\ell)}) - l_2^{(\ell)}(G^{(\ell+1)} - Y^{(\ell)}) + W(Y^{(\ell)}) \in \partial g_1(G^{(\ell+1)}) + \partial I_{\mathcal{C}}(G^{(\ell+1)}). \quad (5)$$

Since g_2 is continuous and convex, we know that $\{W(Y^{(\ell)})\}$ is bounded. By the closeness of ∂g_2, any accumulation point W^* of $\{W(Y^{(\ell)})\}$ belongs to $\partial g_2(Y^*)$. Also, $\{l_1^{(\ell)}\}$ and $\{l_2^{(\ell)}\}$ are bound by definition. Using the continuity of ∇f, there exists a subsequence $\{\ell_k\}$ such that

$$\begin{aligned}
&\lim_{k\to\infty} \Big(\nabla_S f(S^{(\ell_k)}, Y^{(\ell_k)}) + l_1^{(\ell_k)}(S^{(\ell_k+1)} - S^{(\ell_k)})\Big) \\
&= \nabla_S f(S^*, Y^*) \\
&= 0, \qquad (6) \\
&\lim_{k\to\infty} \Big(-\nabla_G f(S^{(\ell_k)}, Y^{(\ell_k)}) - l_2^{(\ell_k)}(G^{(\ell_k+1)} - Y^{(\ell_k)}) + W(Y^{(\ell_k)})\Big) \\
&= \nabla_G f(S^*, Y^*) + W^*. \qquad (7)
\end{aligned}$$

We now show that $g_1(G^{(\ell)}) + I_{\mathcal{C}}(G^{(\ell)}) \to g_1(Y^*) + I_{\mathcal{C}}(Y^*)$. By (3) and (4), we have

$$\begin{aligned}
&\langle \nabla_G f(S^{(\ell)}, Y^{(\ell)}) - W(Y^{(\ell)}), G^{(\ell+1)}\rangle + \frac{l_2^{(\ell)}}{2}\|G^{(\ell+1)} - Y^{(\ell)}\|_F^2 + g_1(G^{(\ell+1)}) + I_{\mathcal{C}}(G^{(\ell+1)}) \\
\le\ &\langle \nabla_G f(S^{(\ell)}, Y^{(\ell)}) - W(Y^{(\ell)}), Y^*\rangle + \frac{l_2^{(\ell)}}{2}\|Y^* - Y^{(\ell)}\|_F^2 + g_1(Y^*) + I_{\mathcal{C}}(Y^*).
\end{aligned}$$

It follows that

$$\limsup_{\ell\to\infty} g_1(G^{(\ell+1)}) + I_{\mathcal{C}}(G^{(\ell+1)}) \le g_1(Y^*) + I_{\mathcal{C}}(Y^*).$$

It is easy to see that g_1 and $I_{\mathcal{C}}$ are lower semicontinuous, i.e.,

$$\liminf_{\ell\to\infty} g_1(G^{(\ell+1)}) + I_{\mathcal{C}}(G^{(\ell+1)}) \ge g_1(Y^*) + I_{\mathcal{C}}(Y^*).$$

Thus,

$$\lim_{\ell\to\infty} g_1(G^{(\ell+1)}) + I_{\mathcal{C}}(G^{(\ell+1)}) = g_1(Y^*) + I_{\mathcal{C}}(Y^*).$$

This, together with (7) and Proposition **??** for (5), yields

$$-\nabla_G f(S^*, Y^*) + W^* \in \partial g_1(Y^*) + \partial I_{\mathcal{C}}(Y^*),$$

i.e.,

$$\begin{aligned}
0 &\in \{\nabla_G f(S^*, Y^*)\} + \partial g_1(Y^*) + \partial I_{\mathcal{C}}(Y^*) - \{W^*\} \\
&\subset \{\nabla_G f(S^*, Y^*)\} + \partial g_1(Y^*) + \partial I_{\mathcal{C}}(Y^*) - \partial g_2(Y^*).
\end{aligned}$$

This, together with (6), implies that $0 \in \partial F(S^*, Y^*)$.

b) Ω_1 is finite. In this case, there exist $L_0 > 0$ such that (2) is satisfied for all $\ell > L_0$. Thus,

$$\sum_{\ell=L_0}^{\infty} (\|S^{(\ell+1)} - S^{(\ell)}\|_F^2 + \|V^{(\ell+1)} - G^{(\ell)}\|_F^2) < \infty,$$

which implies that $\|S^{(\ell+1)} - S^{(\ell)}\|_F \to 0$ and $\|V^{(\ell+1)} - G^{(\ell)}\|_F \to 0$ as $\ell \to \infty$. By following the similar arguments of Case a), for any accumulation point S^* of $\{S^{(\ell)}\}$ and G^* of $\{G^{(\ell)}\}$, we have $0 \in \partial F(S^*, G^*)$.

c) Both Ω_1 and Ω_2 are infinite. In this case, $\|S^{(\ell+1)} - S^{(\ell)}\|_F \to 0$ as $\ell \to \infty$, $\|G^{(\ell+1)} - Y^{(\ell)}\|_F \to 0$ as $\Omega_1 \ni \ell \to \infty$, and $\|V^{(\ell+1)} - G^{(\ell)}\|_F \to 0$ as $\Omega_2 \ni \ell \to \infty$. By hypothesis, both $\{S^{(\ell)}\}$ and $\{G^{(\ell)}\}$ are bounded. Then $\{Y^{(\ell)}\}$ is bounded for all $\ell \in \Omega_1$ and thus $\{\nabla f(S^{(\ell)}, Y^{(\ell)})\}$ is bounded. If follows from Cases a) and b) that, for any accumulation point (S_1^*, Y^*) of $\{(S^{(\ell)}, Y^{(\ell)})\}_{\ell \in \Omega_1}$ and any accumulation point (S_2^*, G^*) of $\{(S^{(\ell)}, G^{(\ell)})\}_{\ell \in \Omega_2}$, we have $0 \in \partial F(S_1^*, Y^*)$ and $0 \in \partial F(S_2^*, G^*)$. We note that $\{G^{(\ell)}\}_{\ell \in \Omega_1}$ and $\{Y^{(\ell)}\}_{\ell \in \Omega_1}$ have the same accumulation point, and $\{V^{(\ell)}\}_{\ell \in \Omega_2}$ and $\{G^{(\ell)}\}_{\ell \in \Omega_2}$ have the same accumulation point. Then, for any accumulation point G^* of $\{G^{(\ell)}\}_{\ell \in \Omega_1}$ and V^* of $\{V^{(\ell)}\}_{\ell \in \Omega_2}$, we have $0 \in \partial F(S_1^*, G^*)$ and $0 \in \partial F(S_2^*, V^*)$. □

References

1. Tono K, Takeda A, Gotoh J, *et al.* Efficient DC algorithm for constrained sparse optimization. arXiv. 2017;arXiv:1701.08498.

2. Li H, Lin Z, *et al.* Accelerated proximal gradient methods for nonconvex programming. In: Adv Neural Inf Process Syst. 2015;379–387.

3. Rockafellar RT, Wets RJB. Variational Analysis. Springer. 1998.

4. Bolte J, Sabach S, Teboulle M, *et al.* Proximal alternating linearized minimization for nonconvex and nonsmooth problems. Math Program. 2014;146(1–2):459–494.
