# Supplementary material for: APDCA: An accurate and effective method for predicting associations between RBPs and AS-events during epithelial-mesenchymal transition
Source: PLoS Comput Biol. 2025 Nov 6;21(11):e1013665. doi: 10.1371/journal.pcbi.1013665 (PMC12604773; doi:10.1371/journal.pcbi.1013665)
Supplement: S2 Text — (PDF) [file pcbi.1013665.s002.pdf]

Data Preprocessing

Data preprocessing

In this study, RNA-binding protein (RBP) expression profiles and exon junction reads were obtained from the TCGA BRCA cohort [1], and known cassette-exon events were retrieved from the MISO [2] database to calculate PSI values using the formula

$$\mathrm{PSI} = \frac{I/L_I}{I/L_I + S/L_S},$$

where I and S denote the inclusion and skipping junction reads, and L_I and L_S represent their effective lengths. Events lacking sufficient junction support in a given sample were treated as missing and excluded from correlation analysis. For each RBP–AS pair, Pearson correlation coefficients were computed across matched samples, and the association matrix was binarized using a threshold of $|r| > 0.5$. The same procedure was applied to construct the RBP–gene and gene–AS association matrices. RBP expression values were directly taken from normalized TCGA data, with all gene identifiers harmonized to HGNC symbols. In addition, heterogeneous biological networks were integrated, including miRNA–gene (miRTarBase [3]), miRNA–disease (HMDD [4]), gene–disease (DisGeNET [5]), gene–drug and drug–drug (DrugBank [6]), and gene–gene interactions (BioGRID). These datasets were uniformly converted into sparse binary adjacency matrices by removing duplicates, harmonizing identifiers, and symmetrizing undirected networks, ensuring consistency and usability across all data sources.

Cross-validation strategy

All performance evaluations were conducted using 5-fold cross-validation on the RBP–AS matrix. In each iteration, the RBP dimension (rows) was randomly partitioned to guarantee mutual exclusivity between training and testing sets, while all auxiliary matrices were kept fixed and used as supplementary information during training. This strategy ensures both fairness and reproducibility of the experimental evaluation.

References

1. Cancer Genome Atlas Network. Comprehensive molecular portraits of human breast tumours. *Nature.* 2012;490:61–70.

2. Katz Y, Wang ET, Airoldi EM, Burge CB. Analysis and design of RNA sequencing experiments for identifying isoform regulation. *Nat Methods.* 2010;7(12):1009–1015.

3. Huang HY, Lin YCD, et al. miRTarBase 2020: updates to the experimentally validated microRNA–target interaction database. *Nucleic Acids Res.* 2020;48(D1):D148–D154.

4. Huang Z, Shi J, Gao Y, et al. HMDD v3.0: a database for experimentally supported human microRNA–disease associations. *Nucleic Acids Res.* 2019;47(D1):D1013–D1017.

5. Piñero J, Ramírez-Anguita JM, Saüch-Pitarch J, et al. The DisGeNET knowledge platform for disease genomics: 2019 update. *Nucleic Acids Res.* 2020;48(D1):D845–D855.

6. Wishart DS, Feunang YD, Guo AC, et al. DrugBank 5.0: a major update to the DrugBank database for 2018. *Nucleic Acids Res.* 2018;46(D1):D1074–D1082.
