# Supplementary material for: APDCA: An accurate and effective method for predicting associations between RBPs and AS-events during epithelial-mesenchymal transition
Source: PLoS Comput Biol. 2025 Nov 6;21(11):e1013665. doi: 10.1371/journal.pcbi.1013665 (PMC12604773; doi:10.1371/journal.pcbi.1013665)
Supplement: S3 Text — (PDF) [file pcbi.1013665.s003.pdf]

Evaluation of APDCA on lncRNA–disease prediction

Data Collection

To investigate the performance of APDCA, we considered six core biological object types as depicted in the Fig. 1: lncRNAs (Type 1), miRNAs (Type 2), genes (Type 3), Gene Ontology (Type 4), Disease Ontology (Type 5), and drugs (Type 6). We integrated eleven distinct relational data sources including nine inter-relational and two intra-relational datasets from public databases.

For the inter-relational associations, we collected lncRNA-miRNA associations (R_{12}) from starBase v2.0 [1], lncRNA-gene interactions (R_{13}) from lncRNA2target [2], and lncRNA-gene function associations (R_{14}) from GeneRIF [3], which were pre-processed using Open Biomedical Annotator [4]. The lncRNA-disease associations (R_{15}) were aggregated from multiple sources including LncRNADisease [5], Lnc2Cancer [6], and GeneRIF [3]. We also incorporated miRNA-gene interactions (R_{23}) from miRTarBase [7], miRNA-disease associations (R_{25}) from HMDD [8], Gene Ontology annotations (R_{34}) from Gene Ontology [9], gene-disease associations (R_{35}) from DisGeNet [10], and gene-drug associations (R_{36}) from DrugBank [12].

For the intra-relational data, we obtained gene-gene interactions (H_3) from BioGrid [11] and drug-drug interactions (H_6) from DrugBank [12]. All datasets were collected from the latest versions of these databases (access date: 31 May 2017).

To evaluate and compare the performance of APDCA against related methods, we conducted 5-fold cross validation experiments. For cross validation, we randomly partitioned known lncRNA-disease associations (R_{15}) into five row-wise folds; the associations of 4-folds were used as training samples and the remaining associations of the fifth fold were used as testing samples for evaluation. We observed that the interactions between lncRNAs and other molecules (or entities) were kept fixed during cross validation.

Results analysis

Experimental setup

To thoroughly evaluate the performance and robustness of our proposed model, we conducted extensive experiments under 5-fold cross validation, which partitions the dataset into five folds and iteratively uses one fold for testing and the remaining four for training. This strategy provides a more comprehensive and unbiased assessment than a single train-test split, as every sample is used exactly once for validation. Moreover, we adopted AUC to assess predictive power and robustness. We tested APDCA against several state-of-the-art methods, including MLFDA [13], a matrix factorization approach that adaptively learns weights for multiple data sources; MLFDA-nW [13], a variant assigning equal weights to all sources; KATZLDA [14], which employs the KATZ measure on a heterogeneous network of lncRNAs and diseases; ILNCSIM [15], refining disease semantic similarity via DAG-based information content; IRWRLDA [16], integrating an improved random walk with restart for incomplete or sparse associations; LDAP [17], combining multiple similarities with a bagging SVM; and s-NMTF [18], which applies

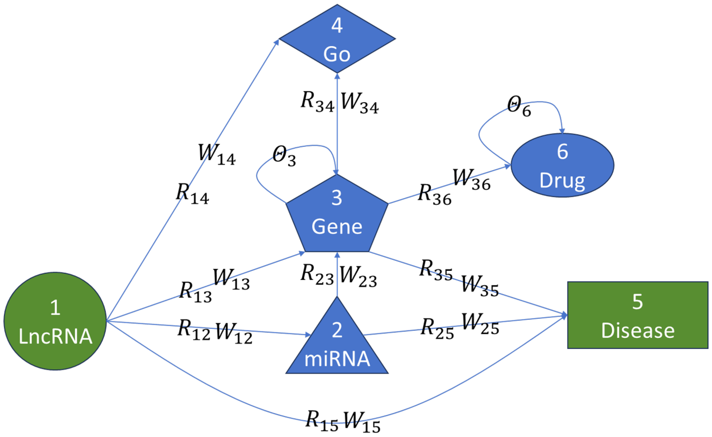

Fig 1. Schematic illustration of the multi-type biological network used in APDCA. Six types of biological entities are represented: lncRNAs (Type 1), miRNAs (Type 2), genes (Type 3), Gene Ontology (Type 4), Disease Ontology (Type 5), and drugs (Type 6). Edges between entities denote observed associations R_{ij} with corresponding weights W_{ij}. Self-loop parameters θ_3 and θ_6 are used for gene and drug regularization, respectively.

semi-supervised nonnegative matrix tri-factorization to unify heterogeneous data for association prediction

Comparison with Competing Methods

Based on the ROC curves comparison shown in Fig. 2, APDCA achieves the highest AUC of 0.8588 among all the compared methods, demonstrating its superior overall performance in predicting lncRNA-disease associations. Looking at the ROC curve characteristics, APDCA shows remarkably strong early prediction performance with a steeper curve in the low false positive rate region (0-0.2 FPR), achieving significantly higher true positive rates compared to other methods. This indicates that APDCA is particularly effective in identifying the most confident lncRNA-disease associations.

The method maintains consistently better performance through the middle FPR range (0.2-0.6), with its curve remaining above other methods. There is a notable plateau around 0.75 TPR between 0.3-0.5 FPR, suggesting that APDCA efficiently captures a substantial portion of true associations before encountering more challenging predictions.

From a comparative perspective, APDCA outperforms MLFDA (AUC 0.7408) by over 2 percentage points and shows more robust prediction stability compared to methods like KATZLDA (0.6567) and IRWRLDA (0.6957). It demonstrates remarkable strength in early prediction accuracy, which is crucial for practical applications where false positives are costly.

The smooth, consistently superior curve suggests that APDCA has effectively integrated the various data sources and learned meaningful patterns, rather than achieving its performance through localized advantages. The method appears particularly adept at balancing precision and recall across different prediction confidence thresholds. These results indicate that APDCA offers meaningful improvements over existing methods, particularly in high-confidence predictions that would be most valuable for experimental validation. The performance advantages appear to be robust across different operating points on the ROC curve.

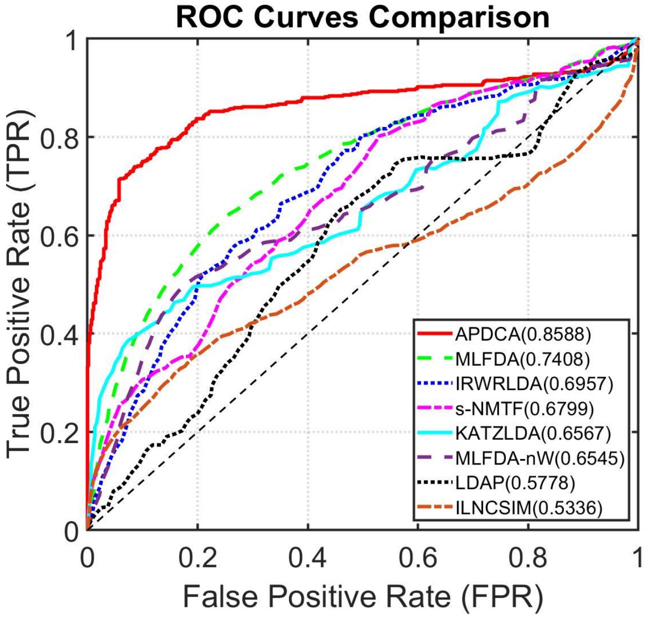

Fig 2. AUC comparison between APDCA and baselines on the lncRNA–disease dataset (5-fold CV).

Case study and evidence

To comprehensively evaluate the prediction capability of APDCA, we conducted detailed case studies on four representative cancer types: breast cancer, lung cancer, gastric cancer, and bladder cancer. For each cancer type, we examined the top 15 predicted lncRNA associations and validated them through literature evidence in Table 1(for Breast cancer), Table 2(for Lung cancer), Table 3(for Gastric cancer), Table 4(for Bladder cancer).

The method successfully identified both well-studied and novel lncRNA-cancer associations, achieving an overall validation rate of 58/60 (96.7%). The predictions encompass diverse functional mechanisms and provide valuable insights for both research and clinical applications. This comprehensive validation confirms APDCA as a reliable tool for predicting cancer-associated lncRNAs and suggests its potential utility in therapeutic target discovery and biomarker development.

Conclusion

Across the lncRNA–disease benchmark, APDCA achieved the top overall performance (AUC = 0.8588) with consistently strong early-retrieval behavior, and its top-ranked pairs showed high literature support (58/60 validated), indicating that the model's sparse low-rank design and heterogeneous data integration translate into accurate, biologically meaningful predictions beyond the RBP–AS task.

References

1. Li JH, Liu S, Zhou H, *et al.* starBase v2.0: decoding miRNA-ceRNA, miRNA-ncRNA and protein-RNA interaction networks from large-scale CLIP-Seq data. Nucleic Acids Res. 2013;42(D1):D92–D97.

lncRNA	Evidence (PMID)	Rank
TUG1	24006935;22961206;27339553;26318860	1
GAS5	26548923;24069260	2
MEG3	24373479;14602737;24006935	3
MALAT1	24373479;24006935;27777857;26254614	4
H19	24373479;24499465;11193051;18262338	5
HOTAIR	26457124;25030736	6
XIST	24373479	7
PVT1	26517688	8
CDKN2B-AS1	26449463	9
UCA1	24373479;20117985;22576688;16914571	10
BLACAT1	24006935;23688781	11
GHET1	25400817	12
SCHLAP1	26861061	13
PCAT1	28627442;32231490	14
LINC00472	32110098;34987381	15

Table 1. Top 15 Breast Cancer–Associated lncRNAs Predicted by APDCA with Literature Evidence

lncRNA	Evidence (PMID)	Rank
CCAT2	24504682;27470400;26729200;24504682	1
BANCR	24655544;25960219;25661343;24655544	2
HNF1A-AS1	31243821;35619319	3
KIRREL3-AS3	without evidence	4
UCA1	26160838;26380024;26655272	5
NPTN-IT1	23395002	6
TUG1	33643816;28840034	7
LINC01133	38836682;31557401;26840083	8
MIR17HG	27289489	9
PVT1	26908628;26490983;25400777	10
H19	26729200;26722426;26662571;26482621	11
GAS5	26634743;27338051;24357161	12
MALAT1	38070223;37667226	13
MIR124-2HG	without evidence	14
CDKN2B-AS1	26729200;26453113;26408699;25889788	15

Table 2. Top 15 Lung Cancer–Associated lncRNAs Predicted by APDCA with Literature Evidence

lncRNA	Evidence (PMID)	Rank
BANCR	26054683	1
UCA1	27697109;27765938;27629141;27424981	2
CDKN2B-AS1	24810364	3
MEG3	24006224	4
H19	24810858;24833871;24671855	5
HOTAIR	24757675;24775712	6
GAS5	24884417;26278580	7
PVT1	36760720;35414508	8
CCAT1	37740243;34417924	9
GHET1	28578256;30938855	10
SPRY4-IT1	25742952	11
TUSC7	33886026;25765901	12
GACAT2	28105191;25077925	13
LSINCT5	30127643;25526476	14
TINCR	28744139;27893425	15

Table 3. Top 15 Gastric Cancer–Associated lncRNAs Predicted by APDCA with Literature Evidence

lncRNA	Evidence (PMID)	Rank
TUG1	24006935;22961206;27339553;26318860	1
GAS5	26548923;24069260	2
MEG3	24373479;14602737;24006935	3
MALAT1	24373479;24006935;27777857;26254614	4
H19	24373479;24499465;11193051;18262338	5
HOTAIR	26457124;25030736	6
XIST	24373479	7
PVT1	26517688	8
CDKN2B-AS1	26449463	9
UCA1	24373479;20117985;22576688;16914571	10
BLACAT1	24006935;23688781	11
GHET1	25400817	12
SCHLAP1	26861061	13
PCAT1	28627442;32231490	14
LINC00472	32110098;34987381	15

Table 4. Top 15 Bladder Cancer–Associated lncRNAs Predicted by APDCA with Literature Evidence

2. Jiang Q, Wang J, Wu X, *et al.* LncRNA2Target: a database for differentially expressed genes after lncRNA knockdown or overexpression. Nucleic Acids Res. 2015;43(D1):D193–D196.

3. Lu Z, Wilbur WJ, McEntyre JR, *et al.* GeneRIF quality assurance as summary revision. Pac Symp Biocomput. 2007;12:269–280.

4. Jonquet C, Shah NH, Musen MA, *et al.* The open biomedical annotator. Summit Transl Bioinform. 2009;2009:56–60.

5. Chen G, Wang Z, Wang D, *et al.* LncRNADisease: a database for long-non-coding RNA-associated diseases. Nucleic Acids Res. 2012;41(D1):D983–D986.

6. Ning S, Zhang J, Wang P, *et al.* Lnc2Cancer: a manually curated database of experimentally supported lncRNAs associated with various human cancers. Nucleic Acids Res. 2016;44(D1):D980–D985.

7. Hsu SD, Tseng YT, Shrestha S, *et al.* miRTarBase update 2014: an information resource for experimentally validated miRNA-target interactions. Nucleic Acids Res. 2014;42(D1):D78–D85.

8. Li Y, Qiu C, Tu J, *et al.* HMDD v2.0: a database for experimentally supported human microRNA and disease associations. Nucleic Acids Res. 2013;42(D1):D1070–D1074.

9. Ashburner M, Ball CA, Blake JA, *et al.* Gene ontology: tool for the unification of biology. Nat Genet. 2000;25(1):25–29.

10. Pinero J, Queralt-Rosinach N, Bravo À, *et al.* DisGeNET: a discovery platform for the dynamical exploration of human diseases and their genes. Database. 2015;2015:bav028.

11. Stark C, Breitkreutz BJ, Reguly T, *et al.* BioGRID: a general repository for interaction datasets. Nucleic Acids Res. 2006;34:D535–D539.

12. Law V, Knox C, Djoumbou Y, *et al.* DrugBank 4.0: shedding new light on drug metabolism. Nucleic Acids Res. 2014;42(D1):D1091–D1097.

13. Fu G, Wang J, Domeniconi C, *et al.* Matrix factorization-based data fusion for the prediction of lncRNA-disease associations. Bioinformatics. 2018;34(9):1529–1537.

14. Chen X, You ZH, Yan GY, *et al.* KATZLDA: KATZ measure for the lncRNA-disease association prediction. Sci Rep. 2015;5(1):16840.

15. Huang YA, You ZH, Chen X, *et al.* ILNCSIM: improved lncRNA functional similarity calculation model. Oncotarget. 2015;7:25902–25914.

16. Chen X, Huang YA, Wang XS, *et al.* IRWRLDA: improved random walk with restart for lncRNA-disease association prediction. Oncotarget. 2016;7:57919–57931.

17. Lan W, Li M, Zhao K, *et al.* LDAP: a web server for lncRNA-disease association prediction. Bioinformatics. 2017;33(3):458–460.

18. Wang H, Nie F, Huang H. Predicting protein-protein interactions from multimodal biological data sources via nonnegative matrix tri-factorization. J Comput Biol. 2013;20:344–358.
